# Lifestyle factors and symptom burden in endometriosis: an exploratory mixed-methods analysis of nutrition, physical activity, and psychosocial well-being

**DOI:** 10.1186/s12905-026-04886-1

**Published:** 2026-09-17

**Authors:** L. Wulfhorst, A. Henze, D. Portius

**Affiliations:** 1 Institute for Agriculture and Nutrition Sciences, Martin-Luther-University Halle-Wittenberg, Halle/Saale, Germany; 2 Personalized Nutrition, Corporative State University Baden-Württemberg, Heilbronn, Germany

**Keywords:** Endometriosis, Lifestyle factors, Dietary habits, Physical activity, Psychosocial well-being, Symptom burden, Mixed-methods study

## Abstract

**Background:**

Endometriosis affects approximately 10% of reproductive-age women worldwide, with symptom burden significantly impacting quality of life. While current treatment guidelines focus primarily on hormonal and surgical interventions, the role of lifestyle factors in symptom management remains underexplored. Major clinical guidelines acknowledge insufficient evidence for specific lifestyle recommendations, highlighting the need for exploratory research. Therefore, our aim is to explore potential associations between lifestyle factors (nutrition, physical activity, psychosocial well-being) and symptom burden in women with endometriosis using a mixed-methods approach.

**Methods:**

A convergent mixed-method design combining a cross-sectional online survey (*n* = 378) with semi-structured expert interviews (*n* = 5) was conducted between January and March 2025. Lifestyle factors were assessed using study-specific composite indices for dietary habits, physical activity, and psychosocial well-being. Symptom burden was evaluated using validated endometriosis-related items informed by established assessment tools and 11-point numeric rating scales for symptom frequency and intensity.

**Results:**

In exploratory analyses, the Dietary Habits Index was not significantly associated with overall symptom burden in the study cohort. However, subgroup analysis revealed a weak negative correlation in participants without food intolerances (β = -0.144, *p* = 0.021). Physical activity and psychosocial well-being indices showed weak associations with symptom burden (physical activity: β = 0.133, *p* = 0.010; psychosocial well-being: β = -0.261, *p* < 0.001). Expert interviews emphasized the importance of individualized, multimodal approaches and highlighted psychosocial factors as particularly relevant for symptom management.

**Conclusions:**

This exploratory study identified weak and context-dependent associations between selected lifestyle factors and endometriosis symptom burden, with psychosocial well-being showing the most consistent relationship. The findings highlight the potential relevance of individualized lifestyle support as an adjunct to conventional medical care. Given the cross-sectional design and methodological limitations, these hypothesis-generating findings require confirmation in longitudinal and intervention studies.

**Supplementary Information:**

The online version contains supplementary material available at https://doi.org/10.1186/s12905-026-04886-1.

## Background

Endometriosis affects approximately 10% of women of reproductive age and is characterized by chronic pelvic pain, fatigue, and infertility, severely affecting quality of life [1, 2]. Despite its high prevalence, endometriosis remains underdiagnosed and poorly understood, with diagnostic delays commonly reported to range between 7 and 10 years [1, 3]. Many women experience persistent symptoms despite hormonal and surgical treatment, leading to a widespread search for complementary strategies to manage (chronic) pain, fatigue, and other persistent symptoms [4, 5].

In recent years, an increasing number of women have adopted lifestyle modifications as complementary strategies to help manage endometriosis-related symptoms and improve quality of life [4]. Surveys from Western populations indicate that approximately 70–80% of women with endometriosis use self-management strategies, including dietary modification, physical activity, and mind–body practices [3, 6, 7]. For example, 75.4% of participants in a survey conducted in Germany, Austria, and Switzerland reported using such strategies, while an Australian survey reported a prevalence of 76% [3, 6, 7]. These approaches are often adopted without professional guidance, highlighting a gap between high patient uptake and structured, evidence-based support for non-pharmacological care.

Emerging research provides biological plausibility for these patient-driven behaviors. Endometriosis is increasingly recognized as a multisystem disease involving reproductive endocrine imbalance, immune dysfunction, neuroinflammation, and psychosocial distress [8, 9]. Aberrant immune activity and systemic inflammation interact with hormonal dysregulation to sustain chronic pain and lesion growth, while psychological stress may further amplify pain sensitivity through neuroendocrine mechanisms [5, 10, 11]. These intertwined biological and psychosocial processes suggest that lifestyle factors that modulate inflammation, anxiety, and emotional well-being may have a meaningful impact on symptom burden and quality of life.

Nevertheless, the scientific evidence supporting lifestyle interventions remains sparse and fragmented. The 2022 European Society of Human Reproduction and Embryology (ESHRE) guidelines identified an urgent need for well-designed studies to assess the efficacy of lifestyle-oriented approaches in endometriosis [12]. Although several small-scale trials have explored dietary interventions, such as anti-inflammatory or Mediterranean-style diets, and mind–body programs, the heterogeneity of study designs, limited sample sizes, and inconsistent outcome measures prevent firm conclusions [13, 14]. Similarly, evidence regarding exercise remains inconclusive. Existing studies suggest potential benefits of moderate, low-impact activity, but the available data are insufficient to support clear clinical recommendations. In contrast, some studies have reported less favorable associations with high-intensity exercise, possibly related to transient increases in pro-inflammatory responses [15–17].

The absence of robust evidence leaves healthcare providers without clear guidance for incorporating lifestyle counseling into the clinical care of endometriosis. At the same time, many patients with endometriosis report substantial symptom relief from personalized combinations of dietary adjustments, physical activity, and stress-reduction or mind–body practices [7, 16, 18]. This paradox between limited empirical data and high patient uptake underscores the need for translational research to bridge the gap between real-world practice and scientific understanding.

Lifestyle interventions are inherently context-dependent, with factors such as comorbidities, hormonal status, psychosocial stress, and individual behaviors potentially influencing their associations with health outcomes. Mixed-methods approaches can therefore complement quantitative estimates by providing insight into the contextual and implementation factors that shape the relevance of lifestyle strategies in clinical practice. Using a convergent mixed-methods design, this study examines associations between nutrition, physical activity, psychosocial well-being, and symptom burden in women with endometriosis and integrates these findings with perspectives from clinical experts. By examining these lifestyle domains simultaneously, we aim to provide a more comprehensive understanding of their potential roles in the complex, individualized management of endometriosis.

## Methods

### Study design and setting

We employed a convergent parallel mixed-methods design, combining quantitative cross-sectional surveys with independent qualitative expert interviews. This approach was selected to capture both measurable associations and contextual insights from clinical experts, thereby enabling triangulation of findings and a more comprehensive understanding of the complex relationships between lifestyle factors and symptom burden. The quantitative component captured patient-reported symptom and lifestyle data, whereas the qualitative component was designed to provide a complementary clinical expert perspective on lifestyle-related aspects and multimodal management of endometriosis.

### Participants and recruitment

For the quantitative component, women aged 18–40 years with a physician-confirmed diagnosis of endometriosis were recruited between January and March 2025. Recruitment was conducted in Germany via online channels, including social media platforms, patient advocacy groups, and endometriosis support networks, enabling nationwide reach and access to individuals actively managing their condition. Inclusion criteria were: (1) medical diagnosis of endometriosis, (2) current experience of endometriosis-related symptoms, (3) age between 18 and 40 years, and (4) sufficient German language proficiency to complete the questionnaires.

Participants were excluded if they were currently pregnant or within one year postpartum, due to potential confounding effects on symptom burden and lifestyle behaviors.

A total of 1181 individuals initiated the survey. After excluding incomplete responses and cases that did not meet the inclusion criteria, 378 participants were retained for the final analysis. The sample size provided sufficient statistical power for exploratory correlation and regression analyses, including subgroup comparisons.

### Quantitative measures

As no existing questionnaire comprehensively captured lifestyle-related factors relevant to the present research question, a study-specific instrument was developed based on the current scientific literature and items informed by established instruments used in health research. Questions relating to endometriosis diagnosis and symptomatology were informed by the *International Endometriosis Evaluation Program 1* (IEEP) [19]. Physical activity items were adapted from the *International Physical Activity Questionnaire* (IPAQ) [20], and dietary items were adapted from the validated food frequency questions of the German Health Interview and Examination Survey for Adults (DEGS1) [21].

The questionnaire comprised 50 core items, with up to 14 conditional filter questions depending on prior responses, to ensure relevance and improve data quality. Items were organized into ten thematic domains: diagnostics, therapy, symptomatology, comorbidities, pregnancy, nutrition, physical activity, psychosocial well-being, lifestyle, and demographic background, including self-reported height and weight. Body mass index (BMI) was calculated as weight in kilograms divided by height in meters squared (kg/m²).

### Overall Symptom Burden Index

Overall symptom burden was assessed using 19 endometriosis-related symptom categories, including pain-related, gastrointestinal, urogenital, cycle-related, and fatigue-related symptoms. Each symptom was assessed separately for frequency and intensity using 11-point numeric rating scales (0–10 in the questionnaire; recoded to 1–11 for analysis), yielding up to 38 symptom ratings. Mean-based composite scores were calculated from the available frequency and intensity ratings, with higher scores indicating greater symptom burden. Missing responses were not imputed. The resulting Overall Symptom Burden Index was a study-specific composite rather than a previously validated endometriosis-specific scale. A complete overview of the included symptoms and their descriptive distributions is provided in Supplementary Tables S8 and S9.

### Lifestyle indices

To capture overarching lifestyle patterns and reduce analytical complexity, lifestyle-related items were aggregated into three composite indices: the Dietary Habits Index (DHI), Physical Activity Index (PAI), and Psychosocial Well-Being Index (PWI). Items were coded in a consistent conceptual direction, such that higher scores represented more favorable dietary habits, greater physical activity, and better psychosocial well-being, respectively. Because the component items of the PAI and PWI were assessed on different response scales, these items were standardized before aggregation. The DHI was constructed from food-frequency items scored on a common food-group-specific metric and therefore did not require item-level z-standardization. For the multivariable and interaction regression models, all three final composite indices were z-standardized before model entry to facilitate comparison across lifestyle domains.

#### Dietary Habits Index

Dietary behavior over the preceding six months was assessed using food-frequency items adapted from the German Health Interview and Examination Survey for Adults (DEGS1), covering the consumption of key food groups (e.g., fruits, vegetables, whole grains, legumes, and processed foods). Response categories in ordinal scores are aggregated to form a composite index. Items were coded such that higher values consistently reflected more favorable dietary patterns. For food groups considered less favorable (e.g., refined grain products, processed foods, fast food, and snack consumption), reverse coding was applied prior to index construction, so that higher consumption corresponded to lower index scores. The resulting Dietary Habits Index represents overall diet quality, with higher scores indicating greater adherence to a health-oriented dietary pattern. A detailed overview of included food groups and coding procedures is provided in Supplementary Table S8. For descriptive and subgroup-specific analyses, the DHI was retained on its original composite scale to preserve interpretability. When included alongside the other lifestyle indices in the multivariable and interaction regression models, the DHI was z-standardized prior to model entry.

#### Physical Activity Index

Physical activity was assessed using items adapted from the International Physical Activity Questionnaire (IPAQ), which capture the frequency, duration, and intensity of both structured exercise and everyday physical activity. Because the component items were assessed using different response scales, they were first coded in a consistent direction, with higher values indicating greater physical activity. Sedentary behavior, such as time spent sitting, was reverse-coded where appropriate. The items were then standardized and aggregated to form the Physical Activity Index (PAI). For regression analyses and graphical presentation, the resulting composite index was additionally z-standardized to facilitate comparison across lifestyle domains. Accordingly, values below zero indicate below-average physical activity and values above zero indicate above-average physical activity relative to the study sample.

#### Psychosocial Well-being Index

Psychosocial well-being was assessed using items capturing perceived stress, emotional burden, relaxation practices, sleep quality, life satisfaction and dimensions of social functioning, including perceived social support and the quality of social interactions. Items were coded in a consistent conceptual direction, with negatively framed items such as perceived stress or emotional burden reverse-coded so that higher values reflected more favorable psychosocial well-being. Because the component items were assessed using different response scales, they were standardized before aggregation into the Psychosocial Well-being Index (PWI). For regression analyses, the resulting composite index was additionally z-standardized, with higher values indicating better psychosocial well-being relative to the study sample.

### Qualitative component

Potential interview participants were identified through search portals of the German Endometriosis Association and the Endo-App. Eligible participants were healthcare professionals with professional experience in the treatment or management of endometriosis. Beginning in December 2024, 11 potential experts were approached by telephone or email and provided with study information. Five experts subsequently participated in the study. The final sample comprised four physicians working in certified or newly certified endometriosis centers or a gynecological practice and one certified nutrition professional specializing in endometriosis.

The five semi-structured interviews were conducted between 11 February and 5 March 2025. Three interviews were conducted in person and two via secure videoconferencing. Interviews lasted, on average, 30 min (range: 18–44 min), were audio-recorded with informed consent, and were transcribed verbatim.

### Data analysis

#### Quantitative analysis

All quantitative analyses were performed using IBM SPSS Statistics, version 27. Descriptive statistics were calculated for sociodemographic and clinical characteristics, symptom burden, and lifestyle indices. Bivariate associations between the Dietary Habits Index (DHI), Physical Activity Index (PAI), Psychosocial Well-being Index (PWI), and overall symptom burden were examined using Pearson and Spearman correlations, followed by simple and multiple linear regression analyses as appropriate. For the multivariable and interaction models, all three lifestyle indices were z-standardized. Unstandardized regression coefficients (b) with 95% confidence intervals (CI) and standardized coefficients (β) were reported. Model assumptions were assessed using standard diagnostic procedures.

Exploratory subgroup analyses were conducted according to self-reported food-intolerance status. For group comparisons, each lifestyle index was divided into three approximately equal categories using the 33rd and 66th percentiles of the respective sample distributions. Differences in symptom burden were assessed using one-way ANOVA with Bonferroni-adjusted post hoc comparisons where appropriate. Additional regression models were conducted to assess the robustness of the associations after adjustment for age and BMI and, separately, for hormonal treatment and surgical history. Exploratory two- and three-way interaction terms between DHI, PAI, and PWI were examined using hierarchical multiple regression.

#### Qualitative analysis

Qualitative data were analyzed using structured qualitative content analysis according to Mayring. Analysis was conducted using MAXQDA 24. A combined deductive–inductive coding approach was applied: initial categories were informed by the research question and theoretical framework, while additional categories were developed from the interview material. The coding framework was reviewed and refined iteratively throughout the analysis to ensure conceptual coherence and consistent application across the dataset.

### Ethical considerations

The study protocol received approval from the Commission for Research Ethics of the Martin-Luther-University Halle-Wittenberg. All participants provided informed consent, and quantitative data were collected anonymously, and qualitative data were pseudonymized to ensure confidentiality. Given the sensitive nature of health-related information and the potential psychological burden of reporting chronic symptoms, participants in the quantitative survey were informed about available support resources.

## Results

### Participant characteristics

A total of 378 women with a physician-confirmed endometriosis participated in the study. The mean age was 30.03 years (SD = 5.48), and the mean BMI was 24.32 kg/m2 (SD = 4.90). Previous endometriosis-related surgery was reported by 77.5% of participants, while 89.7% reported currently using regular medication for endometriosis. Among participants reporting current endometriosis-related medication, commonly reported treatments included analgesics, progestins, oral contraceptives, hormonal intrauterine devices, and GnRH analogues. Common comorbidities included adenomyosis and irritable bowel syndrome. Physician-diagnosed food intolerances were reported by 116 participants (30.7%). Reported types included histamine intolerance (*n* = 38), fructose intolerance (*n* = 23), gluten intolerance (*n* = 20), sorbitol intolerance (*n* = 16), and other intolerances (*n* = 13). Multiple responses were possible. Detailed sociodemographic and clinical characteristics are presented in Table 1.

**Table 1:** Sociodemographic and clinical characteristics of the study population (*n* = 378)

Characteristic	Total sample ***(n = 378)***
Sociodemographic characteristics	
Age, years	30.03 (5.48)
BMI, kg/m²	24.32 (4.90)
Clinical characteristics	
Previous endometriosis-related surgery	293/376 (77.9%)
Current regular endometriosis-related medication	339/376 (90.2%)
Cycle-suppressing medication	207/351 (59.0%)
Adenomyosis	168 (44.4%)
Irritable bowel syndrome	73 (19.3%)
Physician-diagnosed food intolerance	116/375 (30.9%)

Data are presented as mean (SD) for continuous variables and n/N (%) for categorical variables. Denominators vary because of missing responses

*BMI *body mass index

### Quantitative findings

#### Distribution of symptom burden and lifestyle indices

Symptom burden and lifestyle index scores varied substantially across participants, indicating marked heterogeneity in disease impact and lifestyle behaviors. Score distributions spanned a wide range, enabling meaningful exploratory analyses of associations among variables (Table S9).

#### Association between dietary habits and symptom burden stratified by food-intolerance status

**Fig. 1:**
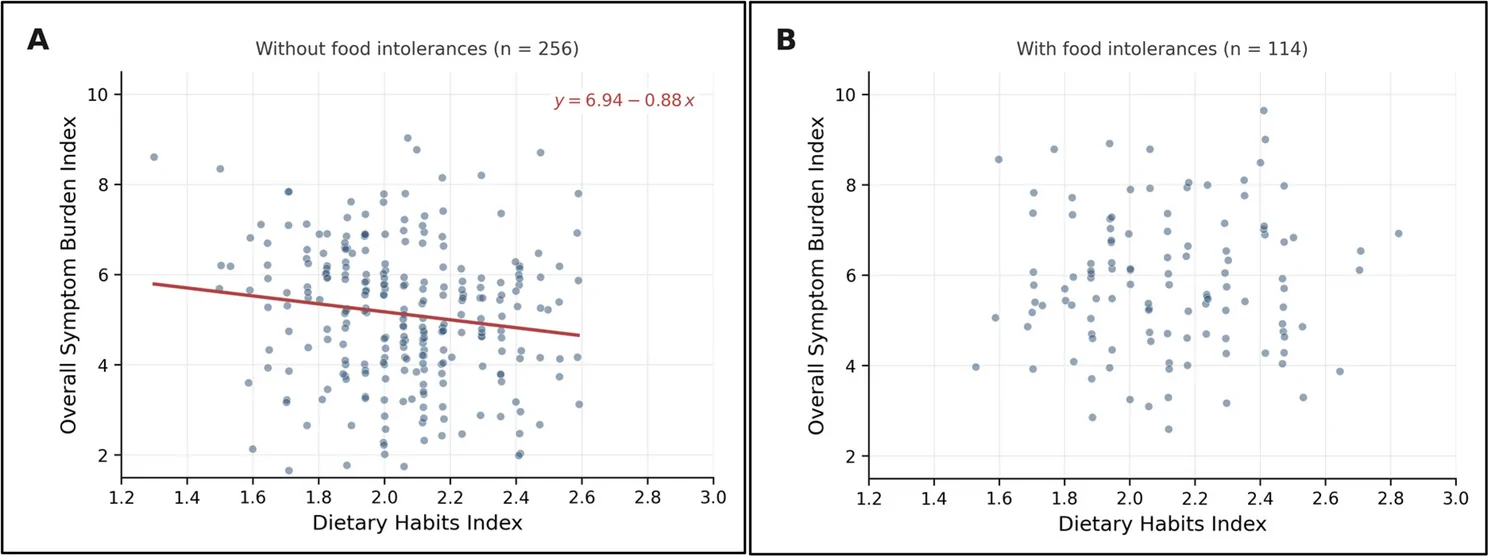
Association between dietary habits and overall symptom burden stratified by food intolerance status. Scatter plots illustrate the relationship between the Dietary Habits Index and the Overall Symptom Burden Index in participants without food intolerances (panel **A**) and with food intolerances (panel **B**). In participants without food intolerances, a weak negative association was identified, indicating lower symptom burden with higher dietary habit scores (Pearson’s *r* = − 0.144, *p* = 0.021; Spearman’s ρ = −0.163, *p* = 0.009; β = −0.144; R² = 0.021; adjusted R² = 0.017). No significant association was observed in participants with food intolerances. Regression lines represent the fitted linear relationship

In the overall sample, the Dietary Habits Index (M = 2.07, SD = 0.26) was not significantly associated with overall symptom burden (*r* = -0.056, *p* = 0.282; ρ = -0.077, *p* = 0.138) (Figure S1). Symptom burden likewise did not differ significantly across low, intermediate, and high dietary habit categories. In exploratory subgroup analyses, a weak inverse association was observed among participants without reported food intolerances (β = -0.144, *p* = 0.021; Table S1; Fig. 1), whereas no association was observed among participants reporting food intolerances (*r* = 0.031, *p* = 0.743; ρ = 0.031, *p* = 0.747).

#### Physical activity is weakly associated with symptom burden

**Fig. 2:**
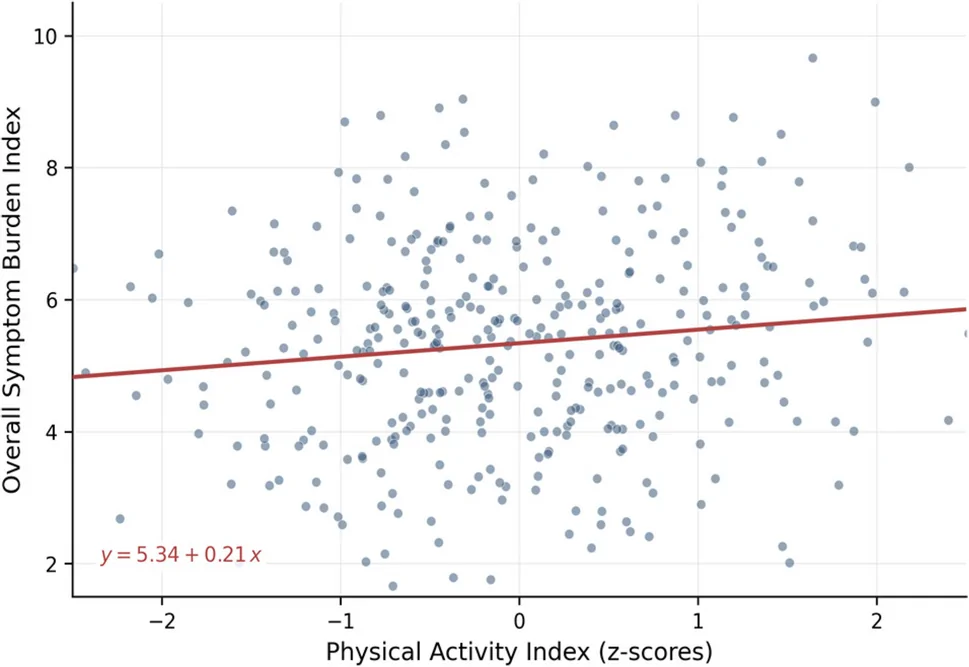
Relationship between Physical Activity Index and overall symptom burden. Scatter plot illustrating the association between the z-standardized Physical Activity Index and the Overall Symptom Burden Index. A weak positive association was observed, with higher Physical Activity Index scores associated with slightly higher symptom burden (β = 0.133, *p* = 0.010; Pearson’s *r* = 0.133; Spearman’s ρ = 0.120). The regression line represents the fitted linear relationship

**Fig. 3:**
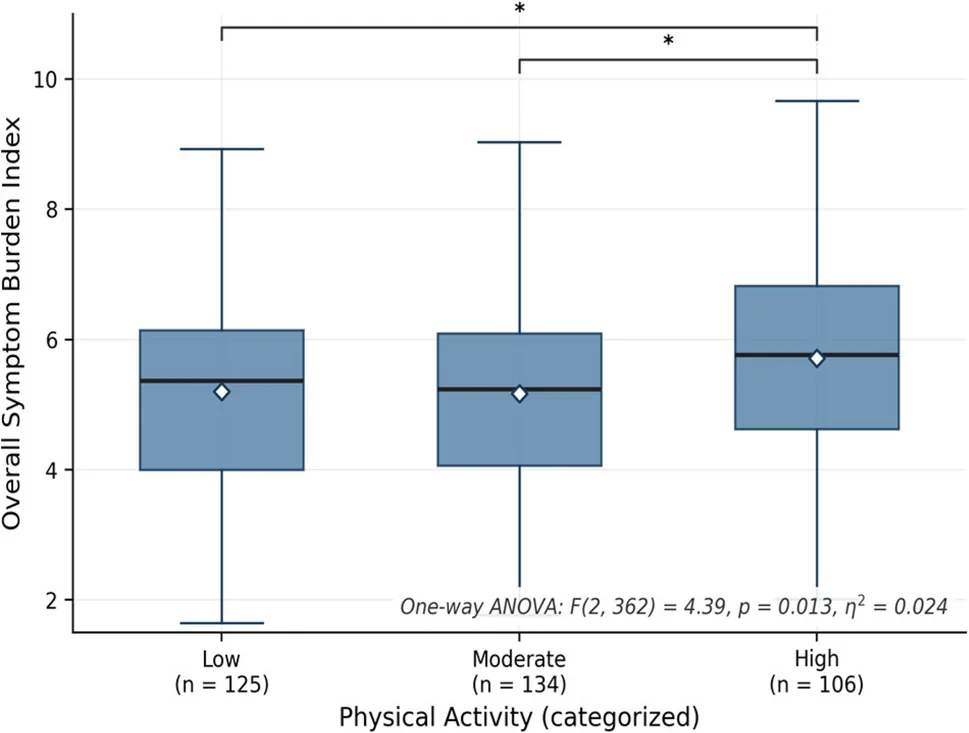
Overall symptom burden across physical activity levels. Boxplots illustrate the distribution of overall symptom burden across low, moderate, and high physical activity groups. A significant overall group difference was observed (ANOVA: F(2) = 4.393, *p* = 0.013, η² = 0.024). Bonferroni-adjusted post hoc comparisons indicated significantly higher symptom burden in the high-activity group than in both the low- and moderate-activity groups, whereas no significant difference was observed between the low- and moderate-activity groups

Overall physical activity levels were weakly positively associated with overall symptom burden (β = 0.133, *p* = 0.010; Table S2; Fig. 2). Symptom burden differed significantly across the low, moderate, and high physical-activity groups (F(2) = 4.393, *p* = 0.013, η² = 0.024). Bonferroni-adjusted post-hoc comparisons indicated significantly higher symptom burden in the high-activity group than in both the low- and moderate-activity groups, whereas no significant difference was observed between the low- and moderate-activity groups (Fig. 3).

#### Higher psychosocial well-being is associated with lower symptom burden

**Fig. 4:**
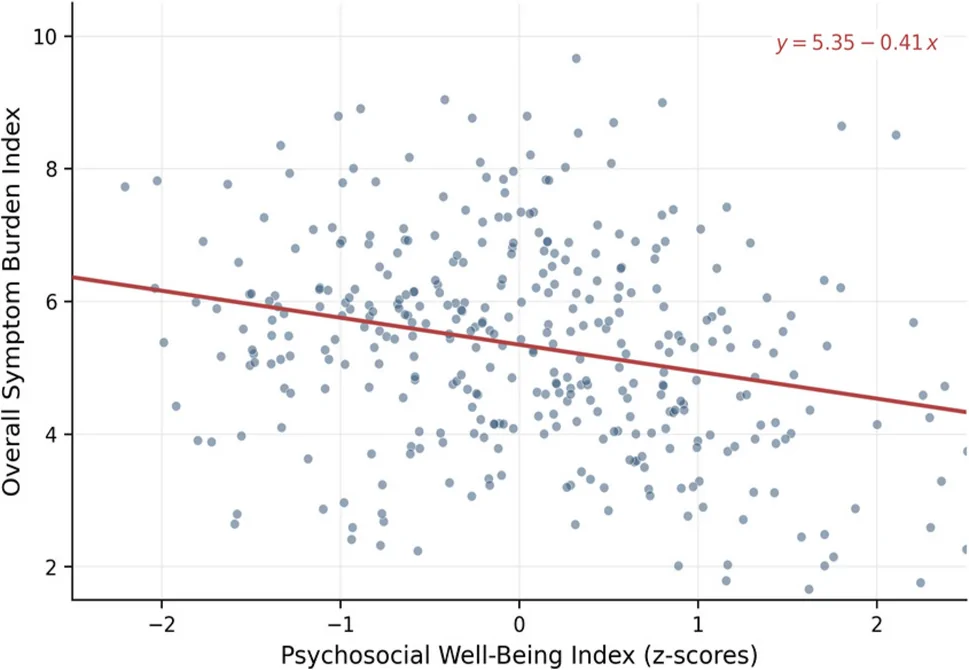
Association between psychosocial well-being and overall symptom burden. Scatter plot illustrating the relationship between the Psychosocial Well-being Index (z-standardized scores) and the Overall Symptom Burden Index. A significant inverse association was observed, indicating lower symptom burden with higher levels of psychosocial well-being (β = −0.261, *p* < 0.001; Pearson’s *r* = − 0.261; Spearman’s ρ = −0.264). The regression line represents the fitted linear relationship

Psychosocial well-being showed the strongest and most consistent association with symptom burden among all lifestyle indices. Higher Psychosocial Well-being Index scores were significantly associated with lower overall symptom burden (β = -0.261, *p* < 0.001; Table S3) (Fig. 4).

Participants with high psychosocial well-being reported significantly less pain interference and better daily functioning than those with moderate or low well-being. These associations remained robust across multiple symptom-related outcome measures.

**Fig. 5:**
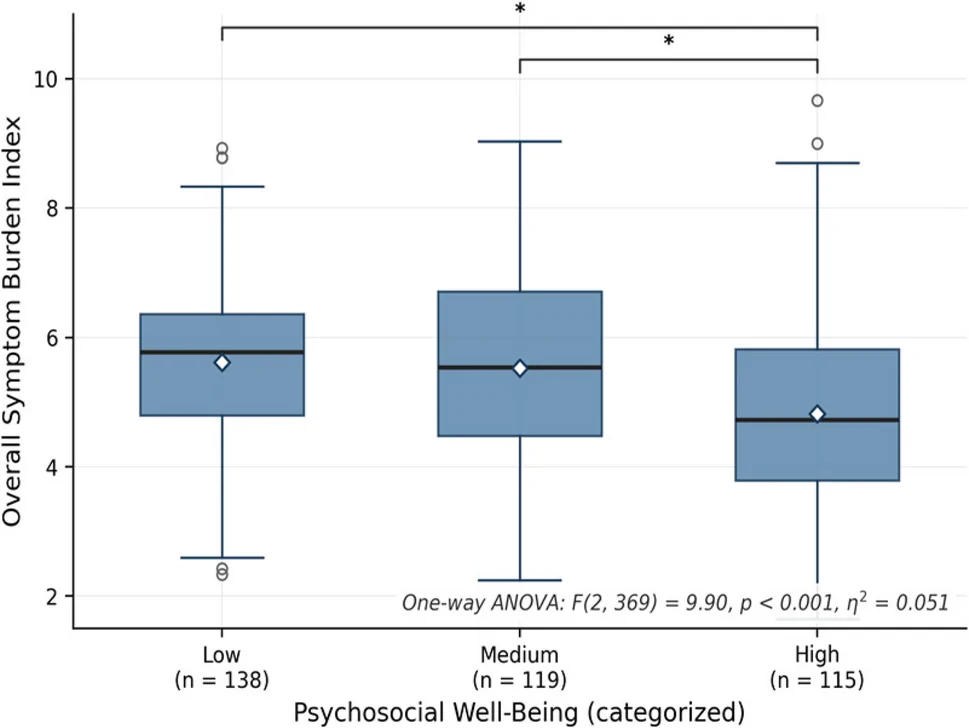
Overall symptom burden across categorized psychosocial well-being levels. Boxplots illustrate the distribution of overall symptom burden in participants with low (*n* = 138), moderate (*n* = 119), and high (*n* = 115) psychosocial well-being. A significant overall group difference was observed (one-way ANOVA: F(2) = 9.904, *p* < 0.001). Post hoc analyses indicated significantly lower symptom burden in the high well-being group compared with both the low (*p* < 0.001) and moderate (*p* = 0.001) groups, whereas no significant difference was observed between the low and moderate well-being groups (*p* = 1.00)

Group comparisons among low, moderate, and high psychosocial well-being demonstrated significant differences (F(2) = 9.904, *p* < 0.001, η² = 0.051), with post hoc analyses indicating that the highest level of psychosocial well-being was associated with the most favorable symptom profiles (Fig. 5).

#### Predictors of symptom burden: multiple linear regression analysis

**Fig. 6:**
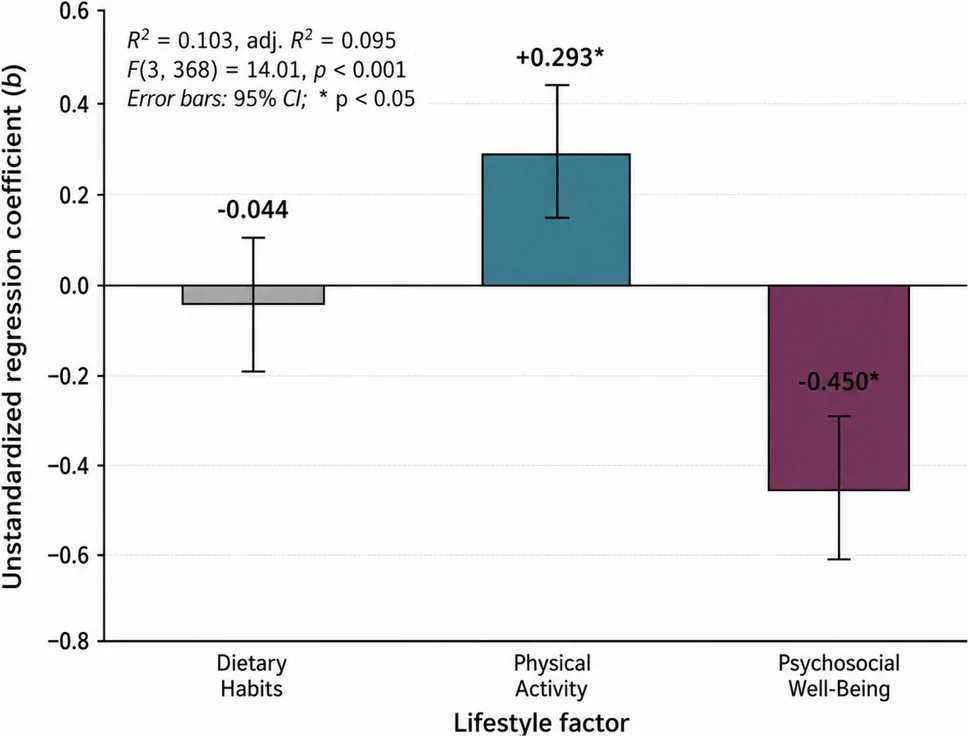
Independent associations of lifestyle factors with overall symptom burden. Unstandardized regression coefficients (b) with 95% confidence intervals from the multiple linear regression model are shown for the z-standardized Dietary Habits Index, Physical Activity Index, and Psychosocial Well-Being Index. Because predictors were z-standardized before model entry, b represents the change in the Overall Symptom Burden Index associated with one-standard-deviation increase in the respective lifestyle index. Psychosocial well-being was inversely associated with symptom burden, whereas physical activity was positively associated with symptom burden. No significant association was observed for dietary habits

In the multiple linear regression model, the Dietary Habits Index was not independently associated with overall symptom burden (b = − 0.044, 95% CI [− 0.200, 0.112], β = -0.028, *p* = 0.581) (Table S4). In contrast, both the Physical Activity Index (b = 0.293, 95% CI [0.139, 0.447], β = 0.190, *p* < 0.001) and the Psychosocial Well-Being Index (b = − 0.450, 95% CI [− 0.607, − 0.294], β = -0.289, *p* < 0.001) were independently associated with symptom burden, with psychosocial well-being demonstrating the largest standardized coefficient (Fig. 6). Adjustment for potential confounders did not materially alter these associations. Including age and BMI as covariates had no meaningful impact on the regression coefficients of the primary predictors. Hormone status and surgical history accounted for a small but statistically significant proportion of variance in symptom burden (R² = 0.021, *p* = 0.020). The addition of the lifestyle indices significantly improved overall model fit, increasing the explained variance to 11.9% (ΔR² = 0.098, *p* < 0.001). No evidence of multicollinearity was observed (all VIFs < 2; tolerance > 0.50).

#### Inter-index correlations and interaction effects

**Fig. 7:**
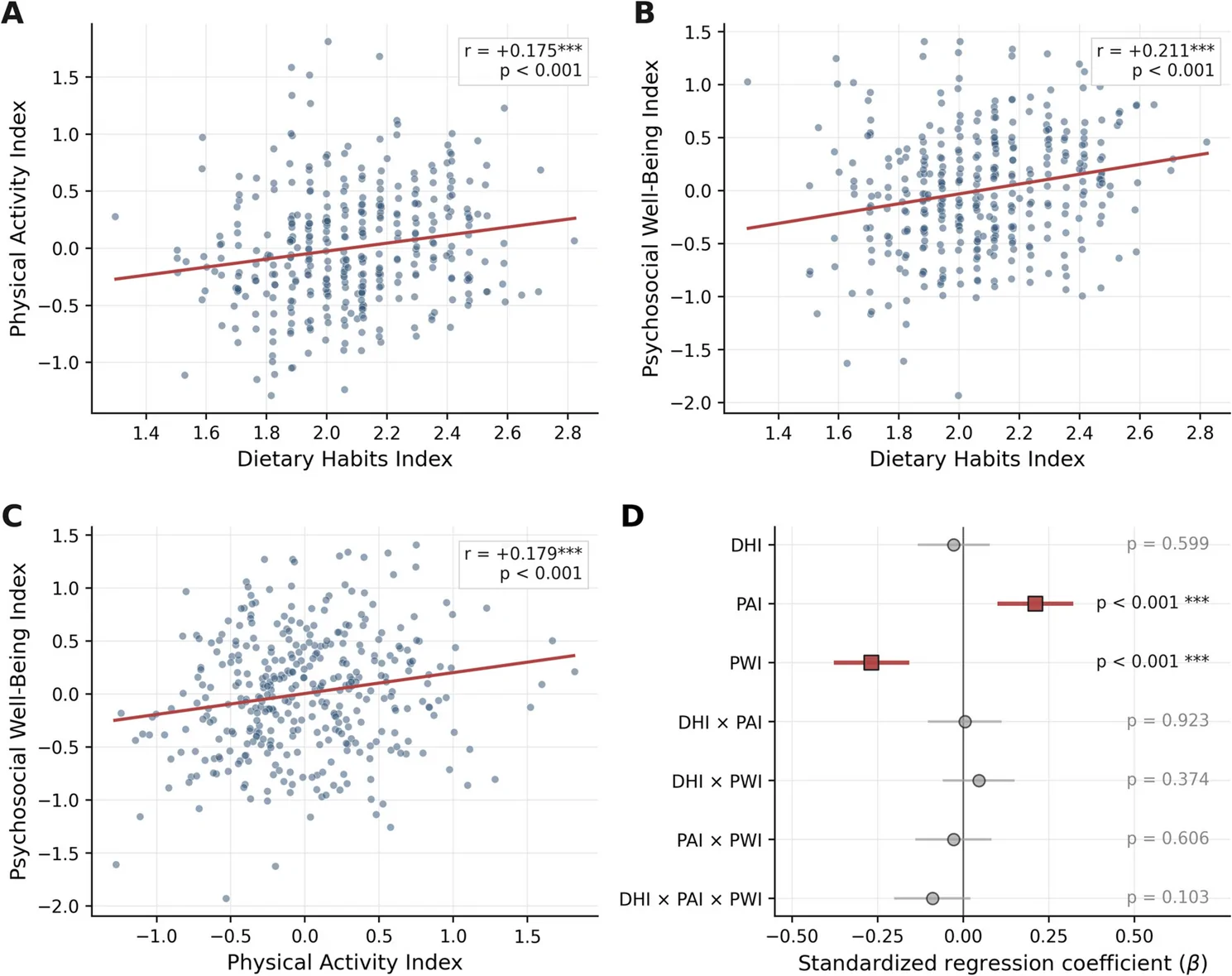
Inter-relationships among lifestyle indices and their interaction effects on overall symptom burden. Pairwise scatter plots illustrate the inter-relationships between the three lifestyle indices in the complete-case sample (*n* = 372): (**A**) Dietary Habits Index (DHI) versus Physical Activity Index (PAI); (**B**) Dietary Habits Index versus Psychosocial Well-Being Index (PWI); (**C**) Physical Activity Index versus Psychosocial Well-Being Index. Red lines represent fitted linear regression; Pearson’s *r* and the corresponding *p*-value are shown in each panel. **D **Standardized regression coefficients (β) with 95% confidence intervals from the full hierarchical interaction model for overall symptom burden. The main-effects model explained 10.3% of the variance (R² = 0.103). Addition of two-way interaction terms did not improve model fit (ΔR² = 0.002, *p* = 0.877), nor did addition of the three-way interaction (ΔR² = 0.007, *p* = 0.103). The full model explained 11.1% of the variance (R² = 0.111). Significant main effects of PAI and PWI were observed, but no two- or three-way interaction term reached statistical significance, indicating no evidence of multiplicative interaction among lifestyle indices. * *p* < 0.05, ** *p* < 0.01, *** *p* < 0.001

To explore whether the three lifestyle indices share common variance and whether their combined effect on symptom burden exceeds the sum of their individual contributions, we conducted exploratory inter-index correlation and interaction analyses (complete-case *n* = 372). All three indices were weakly but significantly positively intercorrelated (DHI–PAI: r = + 0.175, *p* < 0.001; DHI–PWI: r = + 0.211, *p* < 0.001; PAI–PWI: r = + 0.179, *p* < 0.001), indicating modestly overlapping but largely independent lifestyle dimensions (Fig. 7A-C; Table S5). Spearman’s ρ yielded the same pattern (range 0.16–0.22, all *p* < 0.01). A hierarchical multiple regression with z-standardized predictors was then estimated to test whether two- and three-way interactions improved the prediction of overall symptom burden beyond the main-effects model (R² = 0.103; reported above). Adding the three two-way interactions (DHI×PAI, DHI×PWI, PAI×PWI) did not significantly improve model fit (ΔR² = +0.002, F(3, 365) = 0.23, *p* = 0.877), nor did the subsequent inclusion of the three-way interaction (ΔR² = +0.007, F(1, 364) = 2.67, *p* = 0.103). None of the individual interaction terms reached statistical significance (all *p* ≥ 0.10; Fig. 7D; Table S6). No evidence of multiplicative interaction among the lifestyle indices was observed. Psychosocial well-being remained the strongest independent association with symptom burden (β = −0.27, *p* < 0.001), followed by physical activity (β = +0.21, *p* < 0.001), with no significant two- or three-way interaction effects.

### Expert perspectives on multimodal and lifestyle management of endometriosis

The expert interviews were analyzed using a structured qualitative content analysis, yielding 471 coded text segments. These segments were organized into five main categories with 13 subcategories (Table S7), reflecting recurring patterns across interviews. The categories describe experts’ perspectives on (1) disease burden, (2) established therapies, (3) the role of lifestyle factors, (4) multimodal treatment concepts, and (5) implementation conditions in clinical practice.

#### Category 1: disease burden and symptom complexity

Experts consistently described endometriosis as a condition associated with high and multifaceted disease burden. Pain was reported as the dominant symptom, but experts emphasized that pain presentations differed substantially between patients. Described patterns included cycle-related pelvic pain, ovulation-associated pain, and persistent chronic pain independent of the menstrual cycle.

In addition to pain, experts highlighted gastrointestinal symptoms, such as bloating, bowel discomfort, and food-related complaints, as frequent and clinically relevant. Psychological strain, particularly in the context of chronic pain, uncertainty, and infertility, was described as a further dimension contributing to reduced quality of life. Experts noted that the combination of physical and psychological symptoms often resulted in considerable functional limitations in daily life.

#### Category 2: pharmacological and surgical therapies as the foundation of care

Across interviews, experts identified hormonal therapy and surgery as the central pillars of endometriosis treatment. Hormonal therapy was commonly described as a long-term or maintenance strategy aimed at symptom reduction and recurrence prevention, following surgical interventions.

Experts also reported that treatment decisions are frequently shaped by patient preferences, tolerability, and life circumstances. Situations in which hormonal therapy was declined or not tolerated were described as common, requiring alternative or supportive strategies to address ongoing symptoms. Nevertheless, experts consistently framed medical therapies as the foundation of care rather than optional components.

#### Category 3: role of lifestyle factors in symptom management

Lifestyle-related strategies were described as clinically relevant but positioned as adjunctive components, rather than as primary interventions, to support symptom relief and coping. Experts consistently emphasized that lifestyle measures are not a substitute for medical or surgical treatment in patients with confirmed endometriosis.

Experts reported that lifestyle changes were perceived as particularly relevant for symptom domains that are often insufficiently controlled by medical therapy alone. Dietary adjustments were frequently reported in relation to gastrointestinal symptoms, whereas physical activity and movement-based interventions were associated with overall well-being and functional capacity. Experts also noted that patients often attribute high expectations to lifestyle changes, underscoring the importance of clear communication about realistic effects in counseling.

#### Category 4: multimodal treatment as a clinical principle

Rather than describing multimodality as an abstract concept, experts consistently portrayed it as a practical treatment logic. Multimodal care was described as the combination of medical therapy, surgical interventions where indicated, and supportive lifestyle-oriented approaches, adapted to individual symptom patterns and treatment responses.

Experts emphasized that multimodal care is dynamic: treatment components are reassessed and adjusted over time depending on symptom development, patient goals (e.g., fertility intentions), and treatment tolerability. This iterative approach was described as essential for managing this chronic condition, whose symptoms fluctuate.

#### Category 5: structural and contextual factors affecting implementation

Experts identified several factors influencing the implementation of lifestyle-oriented and multimodal care. A central issue was the limited availability of high-quality evidence, which experts perceived as restricting both clinical confidence and broader implementation of lifestyle counseling.

Further constraints included limited consultation time, insufficient reimbursement structures for non-medical interventions, and fragmented care pathways. At the same time, experts highlighted that lifestyle counseling, when feasible, was valued for enhancing patient self-efficacy, as it provides patients with strategies they can actively apply in everyday life. This perceived empowerment was described as an important, albeit indirect, benefit of lifestyle-oriented care.

Overall, the qualitative analysis indicates that experts view endometriosis as a condition characterized by complex symptoms, requiring medical therapy as a foundation, supplemented by lifestyle-oriented measures within a multimodal framework. Lifestyle interventions were consistently described as supportive rather than curative, with their implementation shaped by structural constraints and evidence gaps, yet they were valued for enabling patients to actively engage in day-to-day symptom management and coping.

### Integration of quantitative and qualitative findings

To integrate findings across methodological approaches, quantitative and qualitative results were synthesized to provide a comprehensive understanding of the role of lifestyle factors in endometriosis. Across both data sources, psychosocial well-being emerged as the most consistent and clinically relevant domain, showing the strongest association with symptom burden and being emphasized by experts as central to patients’ everyday experiences and coping processes.

In contrast, associations between physical activity and symptom burden were more modest and appeared to vary depending on activity patterns and individual context. Dietary effects were similarly heterogeneous and were primarily observed in specific subgroups, particularly among women without food intolerances. Importantly, the qualitative findings suggest that these domains should not be interpreted as isolated or independent determinants of symptom burden. Rather, experts consistently described physical activity and dietary strategies as supportive components that may contribute to symptom management, functional capacity, and self-management when integrated into individualized and multimodal treatment approaches.

**Fig. 8:**
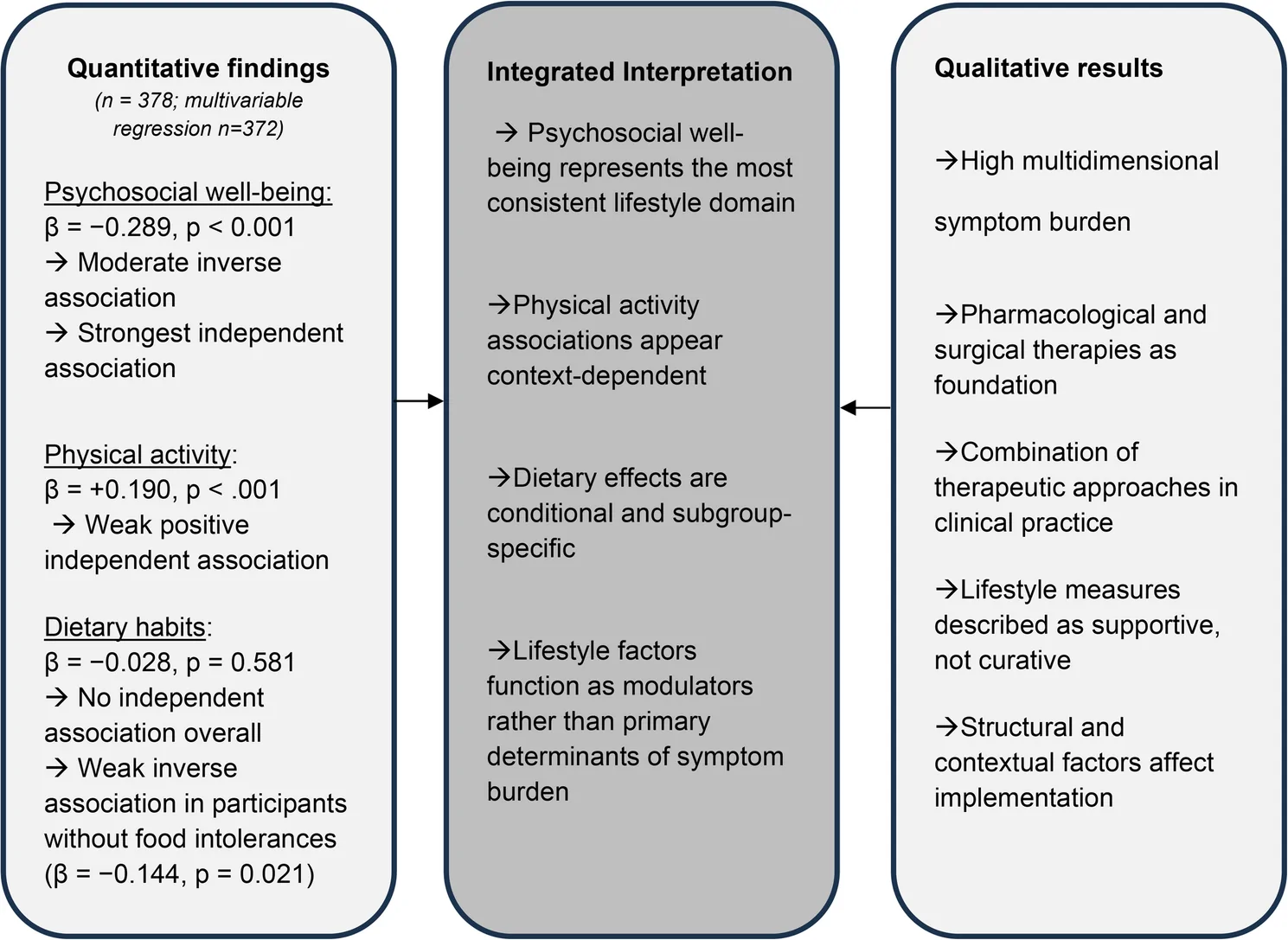
Integrated conceptual model of quantitative and qualitative findings on lifestyle factors and symptom burden in endometriosis. The figure synthesizes quantitative associations with qualitative expert perspectives. Psychosocial well-being showed the strongest independent association, with a weak-to-moderate inverse association with symptom burden, while physical activity showed a weak positive association. Dietary habits were not independently associated with overall symptom burden, although a weak inverse association was observed among participants without reported food intolerances. Qualitative findings positioned lifestyle factors as supportive components within individualized, multimodal endometriosis care

Taken together, the findings indicate that lifestyle-related factors may operate in an interconnected, context-dependent manner rather than exert uniform effects across all patients. While psychosocial well-being demonstrated the strongest and most consistent association with symptom burden, physical activity and dietary behaviors may still play clinically meaningful supportive roles within a broader multimodal framework of endometriosis care. The integrated findings are summarized in Fig. 8.

## Discussion

In this mixed-methods study, we examined associations between lifestyle-related factors and symptom burden in women with endometriosis. Overall, associations between lifestyle factors and symptom burden were generally weak and varied across domains, with psychosocial well-being emerging as the most consistently relevant. However, overlap between psychosocial and behavioral factors should be considered in interpreting these associations. Dietary habits and physical activity showed more differentiated and context-dependent associations. These findings support a nuanced view of lifestyle interventions as adjunctive modulators of symptom experience, rather than primary determinants of disease severity.

Exploratory analyses of the inter-relationships among the three lifestyle indices further refined this interpretation. All three indices were weakly but significantly positively intercorrelated (*r* = 0.18–0.21, all *p* < 0.001; Fig. 7A-C), indicating that women with healthier dietary patterns also tended to report somewhat higher physical activity and better psychosocial well-being. The shared variance between any two indices was small (3–4%), suggesting that the three domains capture largely independent lifestyle dimensions rather than a single underlying construct. Crucially, a hierarchical regression model testing all two- and three-way interactions between the indices revealed no evidence of synergistic effects on symptom burden (combined ΔR² = +0.009 over the main-effects model, all interaction *p* ≥ 0.10; Fig. 7D). The protective association of psychosocial well-being with symptom burden was therefore neither amplified by nor dependent on concurrent dietary or physical activity behaviors. From a clinical perspective, this additive pattern implies that improvements in any single lifestyle domain may contribute meaningfully to symptom relief without requiring simultaneous changes in the others. A finding that may be reassuring for patients facing competing demands when initiating self-management, while also underscoring that no single lifestyle change is sufficient to substitute for the others. Power to detect higher-order interactions in a cross-sectional sample of this size is limited, however, and longitudinal or interventional designs are warranted to test whether combined lifestyle changes produce additive or interactive benefits over time.

Psychosocial well-being demonstrated the strongest and most robust association with symptom burden. Higher psychosocial distress was consistently associated with greater pain interference and reduced daily functioning. This finding aligns with a growing body of evidence indicating that endometriosis profoundly affects mental health and quality of life, with anxiety, depression, chronic stress, and fatigue often matching or exceeding the burden of physical pain itself [22].

Observational studies have repeatedly shown that women with endometriosis report elevated stress levels and that perceived stress correlates with greater symptom severity and functional impairment [22, 23]. The present findings reinforce this evidence by highlighting psychosocial well-being as a key correlate of symptom burden in endometriosis. Importantly, emerging randomized controlled trial evidence further supports the clinical relevance of this association. Meta-analyses of psychosocial interventions, including cognitive-behavioral therapy and mindfulness-based stress reduction, have reported reductions in pain (particularly dyspareunia and dyschezia) and anxiety, alongside improvements in mental health outcomes [23]. Taken together, these findings indicate that psychosocial factors are not peripheral but integral to the symptom experience of endometriosis and support the integration of psychological and stress-regulation strategies into routine care.

Associations between physical activity and symptom burden were modest, with patterns suggesting that the relationship may vary by activity level and type. Overall physical activity levels showed only weak associations with symptom burden, consistent with previous cross-sectional studies reporting limited direct relationships between routine exercise and pain severity [7, 16]. However, group comparisons suggested that symptom burden varied across activity levels. Group comparisons indicated greater symptom burden in the high-activity group than in both the low- and moderate-activity groups, whereas the low- and moderate-activity groups did not differ significantly. These findings indicate that the relationship between overall physical activity and symptom burden may be context-dependent rather than uniformly beneficial across activity levels.

In addition, different forms of physical activity may exert distinct effects. While structured aerobic exercise and strength training may improve physical functioning and overall well-being, movement-based and mind–body approaches, such as yoga or stretching, may also support relaxation, stress reduction, and coping with chronic pain. Evidence from intervention studies nonetheless suggests that exercise can represent a meaningful adjunct in endometriosis care. A recent meta-analysis of six randomized controlled trials (*n* = 251) reported that structured exercise programs improved quality of life and modestly reduced pain [24]. However, considerable heterogeneity in intervention protocols, ranging from aerobic exercise to yoga and stretching, limits conclusions about the optimal type, intensity, or duration of exercise for women with endometriosis.

The qualitative findings provide additional contextual insight into the role of physical activity in the management of endometriosis. Experts emphasized that the context in which physical activity is performed strongly influences its feasibility and perceived usefulness for patients. Exercise approaches that are supervised, individualized, and adaptable to fluctuating symptoms were described as particularly suitable for women experiencing chronic pain and symptom variability. Together, these perspectives suggest that physical activity should not be considered a universally effective intervention, but rather a supportive component of multidisciplinary care when appropriately tailored to individual symptom patterns and functional capacity.

Dietary factors showed no uniform association with symptom burden across the overall cohort. In exploratory subgroup analyses, a weak inverse association between dietary habits and overall symptom burden was observed among participants without reported food intolerances, whereas no association was observed among participants reporting food intolerances. These findings do not indicate that participants with food intolerances experienced greater gastrointestinal symptom burden. Separately, the qualitative interviews suggested that dietary adjustments are frequently considered in relation to gastrointestinal complaints in clinical practice.

Independent of the present subgroup findings, gastrointestinal symptoms are common in women with endometriosis and have been investigated as potential targets for dietary interventions [25]. For example, a recent prospective study reported improvements in gastrointestinal symptoms, quality of life, and emotional well-being among women with endometriosis following a low-FODMAP diet [26]. However, these findings should not be interpreted as evidence that dietary interventions are specifically more effective in women with food intolerances.

Broader nutritional research in endometriosis yields mixed conclusions. While epidemiological studies suggest associations between plant-based, fiber-rich dietary patterns and a lower risk of endometriosis, the overall certainty of the evidence remains low due to observational study designs and potential confounding [27]. A recent narrative review similarly highlighted the potential anti-inflammatory roles of dietary patterns and selected nutrients in endometriosis, while emphasizing that the evidence remains heterogeneous, is frequently derived from preclinical or observational studies, and includes few high-quality human intervention studies [28]. Taken together, the available evidence does not support uniform dietary recommendations and highlights the need for individualized, symptom-oriented approaches. However, the present study did not directly assess whether associations between dietary habits and symptom burden varied by the presence or severity of gastrointestinal symptoms.

Although the observed associations were generally small in magnitude (typically *r* ≈ 0.2–0.3), such effect sizes are expected in a multifactorial condition like endometriosis. Lifestyle factors are unlikely to override the disease’s underlying pathophysiology. However, from a clinical perspective, even small improvements can be meaningful for patients living with chronic pain.

Incremental reductions in symptom burden may translate into improved daily functioning, reduced reliance on analgesics, or enhanced work and social participation. Moreover, lifestyle factors may interact synergistically over time: improved nutrition can enhance energy levels, facilitating physical activity. In turn, regular activity can improve sleep and mood, potentially reducing pain perception. Such cumulative effects may not be fully captured in cross-sectional analyses but are frequently observed in clinical practice [7, 29, 30].

Several limitations should be considered. The cross-sectional design precludes causal inference and limits interpretation of directionality. Lifestyle behaviors and symptom burden may influence each other bidirectionally. The Overall Symptom Burden Index and lifestyle indices were study-specific composite measures and were not independently validated in an endometriosis population, potentially introducing measurement error. Additionally, the qualitative component consisted only of five expert interviews and did not include patient interviews. Therefore, it may not capture the full range of clinical perspectives or provide in-depth insight into patients’ lived experiences, motivations, and perceived barriers to lifestyle modification. Future mixed-methods studies should incorporate qualitative interviews with women living with endometriosis to complement clinical-expert perspectives.

Despite these limitations, the mixed-methods design strengthens the study by enabling convergence between quantitative patterns and qualitative insights. The findings should be interpreted as hypothesis-generating, providing a foundation for future longitudinal and interventional research.

Our findings support integrating lifestyle and psychosocial considerations into endometriosis care alongside established pharmacological and surgical treatments. In practice, this underscores the value of multidisciplinary approaches involving dietitians, physiotherapists, and mental health professionals, particularly for patients with persistent symptoms or specific comorbidities.

Future research should prioritize longitudinal studies and randomized trials to clarify causal pathways, identify patient subgroups most likely to benefit, and determine optimal “doses” and formats of lifestyle interventions. Such studies should prospectively document the timing, duration, and type of complementary or lifestyle-related interventions and examine whether these characteristics are associated with subsequent changes in psychosocial well-being and symptom burden. Continued integration of qualitative methodologies will remain essential to capture patient preferences, feasibility, and contextual factors that quantitative measures alone cannot address.

In summary, this mixed-methods study highlights that lifestyle factors, especially psychosocial well-being, are meaningfully associated with symptom burden in endometriosis, albeit with modest effect sizes. While lifestyle interventions are not curative, they represent valuable adjuncts within a comprehensive, individualized care framework. Addressing psychological well-being, promoting appropriate physical activity, and tailoring dietary advice to symptom profiles may collectively improve quality of life for women with endometriosis.

## Conclusions

In this exploratory mixed-methods study of women with endometriosis, associations between lifestyle factors and overall symptom burden were generally weak and domain-specific. Psychosocial well-being emerged as the most consistent and clinically relevant domain, as physical activity showed a modest, context-dependent association, and dietary habits were related to symptom burden only in the subgroup without food intolerances. Inter-index analyses revealed weak positive correlations between the three lifestyle dimensions but no synergistic interaction effects, indicating that lifestyle factors act largely additively rather than multiplicatively in this population. Expert interviews underscored multimodality and psychosocial care as central to clinical management, while framing dietary and physical-activity strategies as supportive components. These findings support an individualized, multimodal view of lifestyle interventions in endometriosis as adjuncts to pharmacological and surgical care rather than as primary treatments. Given the cross-sectional design and self-reported nature of the data, the results should be considered hypothesis-generating. Longitudinal and intervention studies are warranted to test whether targeted, combined lifestyle changes meaningfully alter the trajectory of symptom burden over time.

## Supplementary Information

Supplementary Material 1.

## Data Availability

The de-identified datasets generated and analysed during the current study, together with the SPSS syntax used for the analyses, are available from the corresponding author on reasonable request.

## Abbreviations

ANOVA: Analysis of variance
BMI: Body mass index
CI: Confidence interval
DEGS1: German Health Interview and Examination Survey for Adults
DHI: Dietary Habits Index
ESHRE: European Society of Human Reproduction and Embryology
IBS: Irritable bowel syndrome
M: Mean
PAI: Physical Activity Index
PWI: Psychosocial Well-Being Index
RCT: Randomised controlled trial
SD: Standard deviation
VIF: Variance inflation factor
