# Supplementary material for: Lifestyle factors and symptom burden in endometriosis: an exploratory mixed-methods analysis of nutrition, physical activity, and psychosocial well-being
Source: BMC Womens Health. 2026 Sep 17;26:461. doi: 10.1186/s12905-026-04886-1 (PMC13584398; doi:10.1186/s12905-026-04886-1)
Supplement: Supplementary file 1 [file 12905_2026_4886_MOESM1_ESM.docx]

**Supplementary Material**

*Wulfhorst L, Henze A & Portius D — Lifestyle Factors and Symptom Burden in Endometriosis: An Exploratory Mixed-Methods Analysis of Nutrition, Physical Activity, and Psychosocial Well-Being*

This file contains the supplementary figures and tables referenced in the main manuscript. Figures are labeled S1–S2 and tables S1–S9 in the order of their first citation in the main text.

# **Supplementary Figures**

**Figure S1.** Association between the Dietary Habits Index and the Overall Symptom Burden Index in the overall sample (n = 372). The fitted regression line is shown in red. No significant association was observed (Pearson’s r = −0.056, p = 0.281; Spearman’s ρ = −0.077, p = 0.137).

**Figure S2.** Overall Symptom Burden Index across categorized Dietary Habits Index levels (low, intermediate, high). The diamond marker denotes the group means; the horizontal bar in the box represents the median. One-way ANOVA did not reveal a significant group difference (F(2, 369) = 0.384, p = 0.681, η² = 0.002).

# **Supplementary Tables**

**Table S1.** Simple linear regression of the Dietary Habits Index on the Overall Symptom Burden Index in participants reporting no food intolerances (n = 256). Dependent variable: Overall Symptom Burden Index. Model: R² = 0.021; adjusted R² = 0.017; F(1, 254) = 5.356, p = 0.021.

| **Coefficient** | **b** | **SE** | **β** | **t** | **p** | **95% CI LL** | **95% CI UL** |
| --- | --- | --- | --- | --- | --- | --- | --- |
| Constant | 6.942 | 0.789 | — | 8.793 | < 0.001 | 5.387 | 8.497 |
| Dietary Habits Index | −0.882* | 0.381 | −0.144* | −2.314 | 0.021 | −1.633 | −0.131 |

*b = unstandardised regression coefficient; SE = standard error; β = standardised regression coefficient; t = t statistic; p = p-value; CI = confidence interval; LL = lower limit; UL = upper limit. All reported p-values are two-tailed. * p < 0.05.*

**Table S2.** Simple linear regression of the (z-standardized) Physical Activity Index on the Overall Symptom Burden Index (n = 372). Dependent variable: Overall Symptom Burden Index. Model: R² = 0.018; adjusted R² = 0.015; F(1, 370) = 6.675, p = 0.010.

| **Coefficient** | **b** | **SE** | **β** | **t** | **p** | **95% CI LL** | **95% CI UL** |
| --- | --- | --- | --- | --- | --- | --- | --- |
| Constant | 5.344 | 0.080 | — | 66.955 | < 0.001 | 5.187 | 5.501 |
| Physical Activity Index (z) | 0.205* | 0.080 | 0.133* | 2.584 | 0.010 | 0.049 | 0.362 |

*Abbreviations as in Table S1. All reported p-values are two-tailed. * p < 0.05.*

**Table S3.** Simple linear regression of the (z-standardized) Psychosocial Well-Being Index on the Overall Symptom Burden Index (n = 372). Dependent variable: Overall Symptom Burden Index. Model: R² = 0.068; adjusted R² = 0.066; F(1, 370) = 27.143, p < 0.001.

| **Coefficient** | **b** | **SE** | **β** | **t** | **p** | **95% CI LL** | **95% CI UL** |
| --- | --- | --- | --- | --- | --- | --- | --- |
| Constant | 5.349 | 0.078 | — | 68.777 | < 0.001 | 5.193 | 5.499 |
| Psychosocial Well-Being Index (z) | −0.407* | 0.078 | −0.261* | −5.219 | < 0.001 | −0.560 | −0.253 |

*Abbreviations as in Table S1. All reported p-values are two-tailed. * p < 0.05.*

**Table S4.** Multiple linear regression of all three z-standardized lifestyle indices on the Overall Symptom Burden Index (n = 372). Dependent variable: Overall Symptom Burden Index. Model: R² = 0.103; adjusted R² = 0.095; F(3, 368) = 14.012, p < 0.001.

| **Predictor** | **b** | **SE** | **β** | **t** | **p** | **95% CI LL** | **95% CI UL** | **Tol.** | **VIF** |
| --- | --- | --- | --- | --- | --- | --- | --- | --- | --- |
| Constant | 5.348 | 0.077 | — | 69.914 | < 0.001 | 5.198 | 5.499 | — | — |
| Dietary Habits Index (z) | −0.044 | 0.079 | −0.028 | −0.553 | 0.581 | −0.200 | 0.112 | 0.936 | 1.068 |
| Physical Activity Index (z) | 0.293* | 0.078 | 0.190* | 3.743 | < 0.001 | 0.139 | 0.447 | 0.948 | 1.055 |
| Psychosocial Well-Being Index (z) | −0.450* | 0.079 | −0.289* | −5.667 | < 0.001 | −0.607 | −0.294 | 0.935 | 1.070 |

*Tol. = tolerance; VIF = variance inflation factor. All VIFs < 2 and tolerance > 0.93, indicating no evidence of multicollinearity. Other abbreviations as in Table S1. All reported p-values are two-tailed. * p < 0.05.*

**Table S5.** Pearson product-moment correlations between the three lifestyle indices in the complete-case sample (n = 372).

|  | **DHI** | **PAI** | **PWI** |
| --- | --- | --- | --- |
| **DHI** | 1.000 | 0.175 (p < 0.001)*** | 0.211 (p < 0.001)*** |
| **PAI** | 0.175 (p < 0.001)*** | 1.000 | 0.179 (p < 0.001)*** |
| **PWI** | 0.211 (p < 0.001)*** | 0.179 (p < 0.001)*** | 1.000 |

*DHI = Dietary Habits Index; PAI = Physical Activity Index; PWI = Psychosocial Well-Being Index. All reported p-values are two-tailed. *** p < 0.001.*

**Table S6.** Full hierarchical multiple regression with two- and three-way interaction terms between z-standardized lifestyle indices predicting the Overall Symptom Burden Index (n = 372). Model fit: R² = 0.111. Adding the two-way interactions to the main-effects model (R² = 0.103) did not significantly improve fit (ΔR² = +0.002, F(3, 365) = 0.23, p = 0.877). Adding the three-way interaction also did not improve fit (ΔR² = +0.007, F(1, 364) = 2.67, p = 0.103).

| **Predictor** | **b** | **SE** | **β** | **t** | **p** |  |
| --- | --- | --- | --- | --- | --- | --- |
| Constant | 5.344 | 0.077 | — | +69.80 | < 0.001 | *** |
| DHI (z) | −0.042 | 0.080 | −0.027 | −0.527 | 0.599 |  |
| PAI (z) | +0.327 | 0.082 | +0.211 | +3.973 | < 0.001 | *** |
| PWI (z) | −0.415 | 0.082 | −0.268 | −5.070 | < 0.001 | *** |
| DHI × PAI | +0.008 | 0.082 | +0.005 | +0.097 | 0.923 |  |
| DHI × PWI | +0.071 | 0.080 | +0.046 | +0.890 | 0.374 |  |
| PAI × PWI | −0.044 | 0.085 | −0.028 | −0.516 | 0.606 |  |
| DHI × PAI × PWI | −0.139 | 0.085 | −0.090 | −1.634 | 0.103 |  |

*Abbreviations as in Table S5 and S1. Interaction terms were computed as products of the z-standardised component indices and then z-standardised before entry into the model. All reported p-values are two-tailed. * p < 0.05.*

**Table S7.** Qualitative coding framework. Main categories, subcategories, and exemplary fine-grained codes derived from the structured content analysis of the five expert interviews.

| **Main category** | **Subcategory** | **Exemplary fine codes** |
| --- | --- | --- |
| 1. Disease burden of endometriosis | Symptomatology; impact of endometriosis | Cycle-dependent lower-abdominal pain; irritable bowel syndrome; endobelly; impairment of daily life |
| 2. Treatment of endometriosis | Hormonal therapy; surgical therapy; lifestyle-oriented therapy | Dietary recommendations; exercise recommendations; relaxation techniques; self-help groups |
| 3. Role of lifestyle factors in treatment | Influence of lifestyle; limits of lifestyle; lifestyle as an additive component | Influence of diet, physical activity, psychosocial factors on symptomatology |
| 4. Multimodality as a therapeutic concept | Combination of multimodal therapy components | Individual, multimodal therapeutic measures |
| 5. Implementation of lifestyle-oriented measures in practice | Resources; challenges; patient compliance; future perspectives | Research funding; interdisciplinary collaboration; insufficient patient information; lack of time; socio-economic costs |

*Five main categories were identified, each further divided into subcategories and illustrated by exemplary fine codes.*

**Table S8.** Items included in the construction of the four indices. Items contributing to the Overall Symptom Burden Index, Dietary Habits Index, Physical Activity Index, and Psychosocial Well-Being Index are listed. Items marked “Recoded” were reverse-scored prior to index calculation so that higher index scores indicate greater symptom burden, more favorable dietary patterns, higher physical activity, and better psychosocial well-being, respectively.

| **Overall Symptom Burden Index** | **Dietary Habits Index** | **Physical Activity Index** | **Psychosocial Well-Being Index** |
| --- | --- | --- | --- |
| 1. Lower abdominal pain during menstruation | 1. Fruits (daily) | 1. Daily activities | 1. Frequency of relaxation techniques (per week) |
| 2. Cycle-independent lower abdominal pain | 2. Vegetables (daily) | 2. Duration of sports (per week) | 2. Sleep quality |
| 3. Chronic pelvic pain | 3. Whole-grain products (daily) | 3. Sport intensity | 3. Quality of social interaction |
| 4. Irregular cycle | 4. Legumes (weekly) | 4. Cycling (per week) | 4. Social embeddedness |
| 5. Spotting/bleeding between periods | 5. Seeds & nuts (weekly) | 5. Walking (per week) | 5. Support from social environment |
| 6. Heavy and/or prolonged bleeding | 6. Fish & seafood (weekly) | 6. Gardening (per week) | 6. Life satisfaction |
| 7. Pain during sexual intercourse | Recoded: | 7. Household chores (per week) | Recoded: |
| 8. Bloating | 7. Refined-grain products (daily) | 8. Occupational physical activity | 7. Stress level |
| 9. Constipation/diarrhoea | 8. Eggs (weekly) | 9. Other physical activities (per week) | 8. Emotional burden caused by endometriosis |
| 10. Pain during defecation | 9. Natural dairy products (weekly) | 10. Step count (per day) |  |
| 11. Pain during urination | 10. Processed dairy products (weekly) | Recoded: |  |
| 12. Urge to defecate | 11. Unprocessed red meat (weekly) | 11. Hours sitting (per day) |  |
| 13. Urinary urgency | 12. Unprocessed white meat (weekly) |  |  |
| 14. Pain in the (lower) back | 13. Processed meat & sausages (weekly) |  |  |
| 15. Chest pain | 14. Ready-made meals (weekly) |  |  |
| 16. Shortness of breath | 15. Fast food (weekly) |  |  |
| 17. Infertility | 16. Sweet snacks (weekly) |  |  |
| 18. Fatigue and exhaustion | 17. Salty snacks (weekly) |  |  |
| 19. Other symptoms |  |  |  |

*Each of the 19 symptom items was assessed separately for frequency and intensity, yielding 38 items contributing to the Overall Symptom Burden Index.*

**Table S9.** Descriptive statistics of symptom frequency and intensity in the study population. Mean (M), standard deviation (SD), and numbers of valid and missing observations are reported for each item. Frequency was rated on a scale from 1 (never) to 11 (always); intensity was rated on a scale from 1 (no intensity) to 11 (maximum intensity). Total sample: n = 378.

| **Symptom** | **Frequency M (SD)** | **n valid** | **n missing** | **Intensity M (SD)** | **n valid** | **n missing**^a^ |
| --- | --- | --- | --- | --- | --- | --- |
| Lower abdominal pain during menstruation | 9.93 (2.08) | 169 | 209 | 8.89 (2.02) | 166 | 212 |
| Cycle-independent lower abdominal pain | 6.69 (2.87) | 375 | 3 | 6.32 (2.59) | 370 | 8 |
| Chronic pelvic pain | 4.51 (3.22) | 368 | 10 | 4.25 (3.04) | 358 | 20 |
| Irregular cycle | 3.90 (3.27) | 167 | 211 | 3.36 (2.98) | 156 | 222 |
| Spotting/bleeding between periods | 3.32 (3.03) | 168 | 210 | 2.88 (2.53) | 158 | 220 |
| Heavy and/or prolonged bleeding | 7.40 (3.60) | 167 | 211 | 6.80 (3.42) | 160 | 218 |
| Pain during sexual intercourse | 6.68 (3.07) | 347 | 31 | 6.12 (2.92) | 342 | 36 |
| Bloating | 7.58 (2.68) | 374 | 4 | 6.87 (2.86) | 364 | 14 |
| Constipation/diarrhoea | 7.18 (2.83) | 375 | 3 | 6.26 (2.78) | 364 | 14 |
| Pain during defecation | 4.78 (3.06) | 374 | 4 | 4.47 (3.04) | 359 | 19 |
| Pain during urination | 3.39 (2.80) | 374 | 4 | 3.18 (2.69) | 360 | 18 |
| Urge to defecate | 3.45 (3.04) | 341 | 37 | 3.08 (2.84) | 320 | 58 |
| Urinary urgency | 4.90 (3.09) | 364 | 14 | 4.12 (2.96) | 353 | 25 |
| Pain in the (lower) back | 7.11 (2.86) | 375 | 3 | 6.42 (2.76) | 366 | 12 |
| Chest pain | 3.35 (2.73) | 372 | 6 | 2.87 (2.44) | 352 | 26 |
| Shortness of breath | 3.21 (2.67) | 373 | 5 | 2.89 (2.47) | 357 | 21 |
| Infertility | 5.69 (4.42) | 167 | 211 | 5.24 (4.46) | 165 | 213 |
| Fatigue and exhaustion | 8.14 (2.36) | 374 | 4 | 7.60 (2.41) | 366 | 12 |
| Other participant-reported symptoms^b^ | 7.40 (3.42) | 113 | 265 | 6.96 (3.40) | 101 | 277 |

*^a^Missing values reflect non-response, items not applicable to the participant (e.g., infertility items shown only to participants who reported attempts at conception), or items below the symptom-burden threshold required for presentation in the questionnaire.*

*^b^“Other symptoms” refers to additional participant-reported symptoms not covered by the 18 predefined symptom categories. These responses were assessed for frequency and intensity as a single “other symptoms” category and were not further subdivided into symptom-specific categories for the present analysis.*
